# Cell-Penetrating Peptides: Emerging Tools for mRNA Delivery

**DOI:** 10.3390/pharmaceutics14010078

**Published:** 2021-12-29

**Authors:** Hidetomo Yokoo, Makoto Oba, Satoshi Uchida

**Affiliations:** 1 Medical Chemistry, Graduate School of Medical Science, Kyoto Prefectural University of Medicine, Kyoto 606-0823, Japan; yokoo@koto.kpu-m.ac.jp; 2 Innovation Center of NanoMedicine (iCONM), Kawasaki Institute of Industrial Promotion, Kawasaki 210-0821, Japan

**Keywords:** mRNA, cell-penetrating peptides, drug delivery systems

## Abstract

Messenger RNAs (mRNAs) were previously shown to have great potential for preventive vaccination against infectious diseases and therapeutic applications in the treatment of cancers and genetic diseases. Delivery systems for mRNAs, including lipid- and polymer-based carriers, are being developed for improving mRNA bioavailability. Among these systems, cell-penetrating peptides (CPPs) of 4–40 amino acids have emerged as powerful tools for mRNA delivery, which were originally developed to deliver membrane-impermeable drugs, peptides, proteins, and nucleic acids to cells and tissues. Various functionalities can be integrated into CPPs by tuning the composition and sequence of natural and non-natural amino acids for mRNA delivery. With the employment of CPPs, improved endosomal escape efficiencies, selective targeting of dendritic cells (DCs), modulation of endosomal pathways for efficient antigen presentation by DCs, and effective mRNA delivery to the lungs by dry powder inhalation have been reported; additionally, they have been found to prolong protein expression by intracellular stabilization of mRNA. This review highlights the distinctive features of CPP-based mRNA delivery systems.

## 1. Introduction

After the identification of the Tat peptide, which is derived from the transcription protein of HIV-1, positions 48–60, a variety of protein-derived or designed cell-penetrating peptides (CPPs), which are peptides with cell membrane permeability, have been developed [1,2]. Some CPPs originate from biomolecules in humans, mice, and viruses. In general, CPPs are internalized by cells via direct cell membrane penetration and/or endocytosis [3,4,5,6,7,8,9]. CPPs have been utilized to deliver membrane-impermeable drugs, peptides, proteins, and nucleic acids to target cells and tissues [7,10,11,12,13], and are expected to be non-viral alternatives to viral vectors [14].

CPPs, including cationic, amphipathic, and hydrophobic peptides, can be flexibly designed to possess various functions for biomedical applications, and can be easily synthesized by reliable methods, such as solid phase peptide synthesis. For example, the addition of histidine (His) residues with a protonated amine with a low p*K*_a_ facilitates the endosomal escape of CPPs. Chemical modification of peptides with non-natural amino acids could preserve secondary structures, such as α-helical structures, resulting in derivatives mimicking the biologically active structures of parent peptides, conferring resistance to enzymatic degradation [15,16,17,18]. Most CPPs contain cationic amino acid residues, such as lysine (Lys) or arginine (Arg). The presence of guanidino groups of Arg residues in CPPs plays a key role in cellular internalization [19,20]; more specifically, guanidino groups interact with anionic groups on the surface of the cell membrane and promote direct penetration of CPPs and/or endocytosis [21,22]. Thus, non-natural amino acids with guanidino groups have been developed as building blocks for CPPs [23,24]. Furthermore, cationic CPPs (for example, Arg-rich CPPs) can also interact with anionic biomacromolecules, such as DNA and RNA, via electrostatic interactions. These properties render CPPs suitable for use as peptide-based vectors.

Although lipids and polymers have been extensively studied in the context of drug delivery systems (DDS), peptides have distinct features. They can be easily and flexibly designed to possess various functionalities, and the composition of each functional unit (amino acid) of peptides can be precisely controlled. In addition, peptides have monodisperse properties, a preferable characteristic of building blocks for synthetic nanoparticles, as they permit control over nanoparticle size and the amount of cargo encapsulated. Moreover, CPPs are typically shorter than cationic polymers, such as polyethyleneimine (PEI), a frequently used polymer for non-viral nucleic acid delivery; thus, CPPs can exhibit considerable cell-penetrating ability with reduced cytotoxicity compared to PEI. Peptide-based nanoparticles can be easily synthesized in a single-step process. These potentials are helpful for overcoming the limitations encountered during the clinical translation of conventional DDS nanoparticles, such as short circulation half-lives, uncontrolled biodistribution, and poor bioavailability. Therefore, this approach is further expected to expand the options of delivery routes, including oral delivery [25]. 

## 2. CPPs for Nucleic Acid Delivery

CPPs have been widely used for the delivery of nucleic acids, including plasmid DNA (pDNA), siRNA and antisense oligonucleotide (ASO), demonstrating their huge potential [26], whereas a limited number of reports have addressed the application of CPPs for mRNA delivery. Prior to a detailed description of mRNA delivery in the next section, this section provides an overview of CPP application to nucleic acid delivery, referring to research on pDNA, siRNA, and ASO delivery. CPPs facilitate the cellular uptake of these nucleic acids, which are impermeable to cellular and endosomal membranes in their naked forms because of their large molecular sizes and anionic charges. In addition, complexation of nucleic acids with CPPs helps prevent nuclease-mediated degradation before and after cellular uptake. CPPs are either covalently or non-covalently conjugated to nucleic acids. The covalent approach is especially useful for neutrally charged nucleotide analogs and non-cationic CPPs [27]. In most cases, nucleic acids are anionically charged and spontaneously complexed with cationic CPPs through electrostatic interactions upon physical mixing [7,28,29]. This non-covalent approach provides a simple and robust method for conjugation of CPPs and nucleic acids and is thus mainly employed in the delivery of nucleic acids. CPP/pDNA and CPP/siRNA complexes allow the efficient introduction of these nucleic acids in vitro and in vivo, showing therapeutic feasibility in several disease models, including various types of cancer [26].

CPPs have multiple functions in CPP/nucleic acid complexes, such as protecting nucleic acids from nucleases, enhancing cellular uptake, and targeting specific cells. In addition to direct complexation of CPPs and nucleic acids, CPPs are utilized to target nanoparticles loaded with nucleic acids to specific organs and cells. Cellular uptake and endosomal escape are facilitated by attachment of CPPs to the surface of the nanoparticles. Lipid-based carriers, which are typically used in this strategy, can load a large number of nucleic acids, protect nucleic acids from nucleases, and allow for a prolonged circulation time in blood. For example, siRNA delivery vectors targeting dendritic cells (DCs) have been developed by surface modification of a multifunctional envelope-type nanodevice (MEND) with GALA, a CPP that enhances endosomal escape, which possesses an oligoarginine/siRNA core surrounded by a lipid membrane [30]. In an in vivo experiment, nanoparticles composed of MEND and siRNA modified with GALA induced efficient gene silencing in the pulmonary endothelium [31]. CPPs have also been vigorously studied for the delivery of ASO to modulate pre-mRNA, showing promising outcomes in the treatment of neuromuscular disorders including Duchenne muscular dystrophy [32].

## 3. CPPs for mRNA Delivery

### 3.1. Concepts for the Use of CPPs in mRNA Delivery

Messenger RNAs (mRNAs) have emerged as attractive modalities for nucleic acid-based therapeutics. Two types of mRNA vaccines have been approved for coronavirus disease 2019 (COVID-19) [33]. Apart from preventive vaccines against infectious diseases, mRNA-based approaches have been demonstrated to have a huge potential in therapeutic settings, based on studies in animal models and clinical trials, including cancer vaccination and immunotherapy, genome editing, and the supplementation of deficient proteins in genetic disorders [34,35,36]. For preventive and therapeutic applications of mRNA, delivery carriers are used to protect the mRNA from ribonucleases (RNases) and to alleviate their immunogenicity. In this regard, systems based on lipids and polymers have been extensively studied and reviewed elsewhere [37,38,39,40]. Considering the outstanding potential of CPPs in nucleic acid delivery, which has been described above, CPPs are promising carriers for mRNA delivery, either as CPP/mRNA complexes or in combination with other nanoparticles. 

A pioneering study in 2001 revealed the potential of short cationic peptides for mRNA delivery [41]. In this study, mRNA was complexed with short poly(L-Lys) (PLL) (3.4 kDa) and long PLL (54 kDa). While the strong association of long PLL with mRNA in the cytoplasm inhibited the translation of mRNA, complexation with short PLL facilitated the smooth release of mRNA in the cytoplasm, highlighting its potential utility in mRNA delivery. However, short PLL lacks the endosomal escape capability, thereby providing low protein expression efficiency from mRNA in cultured cells in the absence of endosomal escape reagents, such as chloroquine. In the same study, the effect of polycation length on mRNA introduction capabilities was also investigated using branched PEI (bPEI) with two different lengths. Like PLL, short bPEI (2 kDa) smoothly released the mRNA in the cytosol, but failed to induce endosomal escape, while the long variant (25 kDa) inhibited mRNA translation in the cytosol. Conjugation of melittin, a natural CPP, to bPEI enhanced the endosomal escape of the mRNA, presumably by disrupting the endosomal membrane and improving protein expression efficiency, thus providing proof of concept for the use of CPPs in mRNA delivery. Thus, CPPs for mRNA delivery should be designed to disrupt the endosomal membrane and bind to mRNA with moderate strength such that ribosomal binding is not compromised and efficient translation takes place in the cytosol.

**Figure 1:**
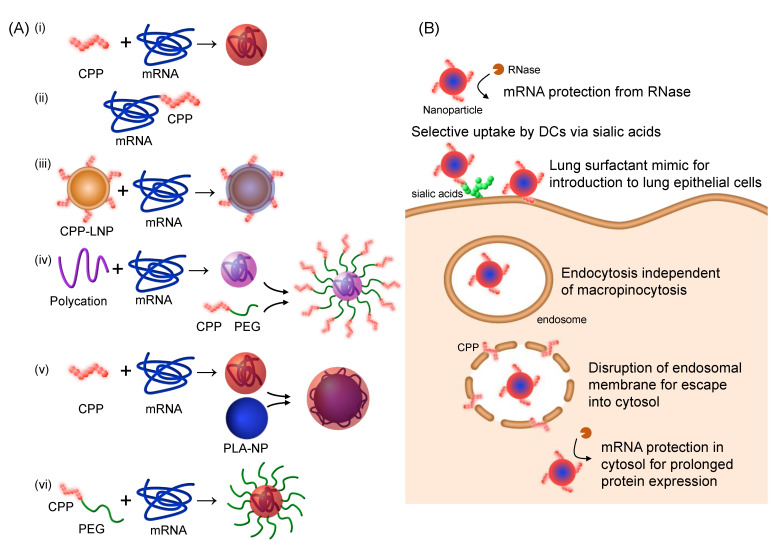
Cell-penetrating peptides (CPPs) for mRNA delivery. (**A**) Preparation of CPP-based mRNA nanoparticles. (**i**) Physical mixing of CPPs and mRNA. (**ii**) Covalent conjugation of CPP to mRNA. (**iii**) Lipoplex preparation from CPP-introduced lipid nanoparticle (LNP) and mRNA. (**iv**) Post-conjugation of poly(ethylene glycol) (PEG)-CPP to mRNA and polycation polyplexes via click reaction for presenting CPPs on the surface. (**v**) Coating of poly(lactic acid) (PLA) nanoparticles with CPP/mRNA complexes. (**vi**) Physical mixing of PEG-CPPs and mRNA for preparation of PEGylated nanoparticles. (**B**) Functionalities of CPPs in mRNA delivery.

The following studies mainly utilized the endosomal escape capability of CPPs in mRNA delivery, either as CPP/mRNA complexes or in combination with other nanoparticles (Figure 1A). Some CPPs selectively targeted DCs, while others exhibited promising outcomes after in vivo delivery (Figure 1B). In addition to the use of CPPs as endosomal escape-promoting reagents, several studies have shown CPPs to have other functionalities, for instance, facilitating mRNA uptake into lung cells after aerosol administration or prolonging the translation of mRNA. The following sections describe the applications of CPPs in mRNA delivery in more detail, with a summary of the systems listed in Table 1.

### 3.2. Enhanced Cellular Uptake and Disruption of Endosomal Membrane

In many settings, CPPs have been used for facilitating cellular uptake and the endosomal escape of mRNA, and the physical mixing of anionic mRNA and cationic CPPs is a straightforward method to non-covalently conjugate CPP to mRNA. De Koker et al. utilized an amphipathic CPP, RALA, which is composed of cationic Arg, as well as hydrophobic alanine (Ala) and leucine (Leu) residues, to develop mRNA vaccines (Table 1) [42]. Nanoparticles below 100 nm were prepared by mixing RALA and mRNA (Figure 1(Ai)). Based on the presence of His and glutamate residues, RALA selectively disrupts the cell membrane at endosomal pH, inducing efficient endosomal escape of the RALA/mRNA complex. This complex was shown to evoke efficient cellular immunity after intradermal injection into mice using a model antigen. Intriguingly, by introducing nucleoside modifications using pseudouridine and 5-methylcytidine, the vaccination effect of the RALA/mRNA complex was greater than that of a standard liposomal mRNA formulation composed of the cationic lipid 1,2-dioleoyl-3-dimethylammonium-propane (DOTAP) and the fusogenic lipid 1,2-dioleoyl-sn-glycero-3-phosphoethanolamine (DOPE). In an experiment addressing the underlying mechanism, cationic liposomes induced type I interferon expression even after introducing nucleoside modifications, which may have dampened the vaccination effect, while nucleoside modification in the RALA/mRNA complex effectively suppressed type I interferon responses. Thus, the low inflammatory nature of RALA peptides may contribute to the efficiency of the vaccination. The importance of the RALA sequence was also studied using two control peptides: the RGSG peptide, in which the hydrophobic Ala and Leu residues in RALA were replaced with hydrophilic glycine and serine residues, and the RRRR peptide, which is rich in Arg, but contains few hydrophobic amino acids (Table 1). These control peptides showed inefficient endosomal escape capabilities and failed to induce a vaccination effect in mice.

Brock et al. used an amphipathic CPP with N-terminal stearylation, PepFect 14 (PF14), to deliver mRNA in tissues from patients, cell spheroids and mouse models of epithelial ovarian cancer (EOC), one of the most lethal gynecological malignancies (Table 1) [43]. PF14/mRNA complexes were prepared by physical mixing (Figure 1(Ai)). Although PF14 induced lower mRNA introduction efficiency in cultured cells compared with a commercially available lipid-based reagent, only PF14 provided detectable protein expression after intraperitoneal injection into a mouse model of peritoneally disseminated ovarian cancer. Interestingly, the PF14/mRNA complex was selectively distributed to the tumor, with almost undetectable accumulation in the abdominal organs, including the liver, spleen, and kidneys. Inside the tumor tissues, it induced protein expression in both cancer and non-cancer cells, including fibroblasts and immune cells. This complex also exhibited successful mRNA introduction into human tumor explants. Meanwhile, protein expression from the PF14/mRNA complex was limited to the surface of the tumor tissue in both a mouse model of cancer and human tumor explants, as well as after in vitro transfection into three-dimensional cancer cell spheroids, despite the tissue-penetrating properties of PF14. The size of the PF14/mRNA complex was determined to be approximately 100 nm, which may have been too large for tissue penetration. Together, these two studies clearly demonstrated the in vivo utility of CPP/mRNA non-covalent complexes, although further optimization is needed for clinical translation.

Covalent conjugation of CPP to mRNA has also been reported. Miliotou et al. conjugated a neutral hydrophobic peptide, PFVYLI, to mRNA, by ligating mRNA with a phosphorylated puromycin–PFVYLI conjugate (Figure 1(Aii), Table 1) [44]. The functionalities of this system were evaluated in vitro without the use of cationic delivery components. PFVYLI conjugation improved the nuclease stability of mRNA compared with that of non-conjugated mRNA and facilitated the cellular uptake of mRNA compared with that of mRNA conjugated with a control peptide, WSYGLRPG. The therapeutic potential of this system for genetic disorders was evaluated by introducing mRNA encoding deficient genes to the cultured cells derived from patients with two types of disorders: cytochrome c oxidase (COX) deficiency caused by an *SCO2* mutation and β-thalassemia caused by a *β-globin* mutation. Introduction of *SCO2* or *β-globin* mRNA provided detectable expression of these deficient proteins in the patient cells for at least 4 days.

Combinations of CPP with lipid-based nanoparticles have also been pursued in mRNA delivery. Tateshita et al. developed lipoplex-based mRNA vaccines from CPP-installed lipid nanoparticles (LNPs) and mRNA (Figure 1(Aiii), Table 1) [45]. LNPs from ionizable lipids with vitamin E scaffolds, phospholipid, and cholesterol were mixed with stearyl CPP to introduce CPP onto the LNP, with KALA, α-helical cationic peptide, used as a CPP, and with mRNA to prepare lipoplexes. Compared with control lipoplex introduced with octaarginine (R8, Table 1), the KALA-lipoplex improved cellular uptake efficiency and hemolytic activity at pH 5.5, which represents the ability of endosomal escape. Intriguingly, KALA-lipoplex loading mRNA induced efficient expression of proinflammatory cytokines in bone marrow-derived DCs (BMDCs), demonstrating its functioning as an immunostimulatory adjuvant for vaccines. As empty KALA-lipoplex failed to exhibit proinflammatory responses, the adjuvant effect of mRNA KALA-lipoplex may be attributed to the enhanced presentation of mRNA onto intracellular innate immune receptors. After the introduction of mRNA encoding ovalbumin (OVA), a model antigen into BMDCs, KALA-lipoplex induced enhanced presentation of an OVA epitope in major histocompatibility complex (MHC) class I compared to that after R8-lipoplex introduction. Ultimately, transplantation of BMDCs introduced with *OVA* mRNA using KALA-lipoplex provided efficient cellular immunity and anti-cancer effects in mice inoculated with OVA-expressing tumor, demonstrating the utility of the mRNA lipoplex for ex vivo cancer vaccination.

### 3.3. Modulation of Endocytotic Pathways in DCs

Interestingly, CPPs are capable of modulating endocytotic pathways of mRNA nanoparticles, presumably via binding to specific cell surface molecules. Two independent reports addressed this point and achieved efficient mRNA introduction to DCs by avoiding a specific endocytotic pathway that is downregulated in mature DCs. For targeting cell surface molecules, CPPs are preferably presented on the surface of nanoparticles, which is effectively achieved by post-conjugation of CPP to nanoparticles [50]. Mastrobattista et al. employed this approach to present GALA peptides on the surface of mRNA nanoparticles to introduce mRNAs into DCs (Table 1) [46]. After preparation of polyplexes from polycations and mRNA, poly(ethylene glycol) (PEG)-GALA was conjugated to the polyplex via click reaction to prepare GALA-modified mRNA polyplexes (PPx/GALA) (Figure 1(Aiv)). PPx/GALA displayed a size of 350–400 nm and was negatively charged because of the negative charge of the GALA moiety. PPx/GALA modulated the endocytosis pathway in DCs to maximize antigen presentation. While macropinocytosis is predominant in the uptake of lipid-based mRNA nanoparticles by DCs, this pathway is downregulated after DCs have matured via the self-adjuvating effect of mRNA or other mechanisms [51,52]. Although nucleoside modifications can suppress DC maturation, it is required for effective vaccination [53]. Notably, PPx/GALA was internalized by DCs via receptor-mediated endocytosis, during which the GALA moiety may have bound to sialic acid-terminated glycans on the DCs. Utilizing receptor-mediated endocytosis instead of micropinocytosis, PPx/GALA was internalized more efficiently than a lipid-based system by mature DCs. After endocytosis, GALA disrupted the endosomal membrane through its pH-sensitive fusogenic properties [54,55] to facilitate endosomal escape, thereby providing more efficient reporter protein expression in macrophages and DCs compared to a commonly used lipid-based system, without eliciting any noticeable cytotoxicity. After the introduction of antigen mRNA into DCs, PPx/GALA outperformed the lipid-based reagent in terms of epitope presentation efficiency at MHC class I molecules and DC activation, revealing its utility as an mRNA vaccine. Importantly, PPx/GALA induced only modest mRNA introduction into non-immune cells, such as HEK293T cells. Immune cell tropism was diminished when GALA was replaced with melittin, a cationic and hemolytic peptide, and LEDE, an antimicrobial cationic peptide (Table 1).

Biodegradable nanoparticles based on a poly(lactic acid) (PLA) backbone have recently gained interest in vaccine development, as they facilitate the efficient introduction of antigens into DCs in vitro and in vivo, providing a safe strategy for inducing vaccination effects [56,57,58,59,60,61]. For the application of PLA nanoparticles (PLA-NPs) to mRNA delivery, negatively charged PLA-NPs should be loaded with negatively charged mRNA biomolecules and also possess endosomal escape capabilities. The use of CPPs as cationic intermediates with the ability to induce membrane disruption is a promising approach, as all requirements are met [41]. Verrier et al. screened three types of amphipathic CPPs: LAH4 and LAH4-L1 peptides, which are comprised of cationic Arg, pH-responsive His, and hydrophobic Ala and Leu residues [47], and RALA peptide, which is composed of cationic Arg and hydrophobic Ala and Leu residues (Table 1, see Section 3.2 for details). Of note, LAH4 and LAH4-L1 peptides have the same amino acid composition and differ only in their sequence alignment. After mixing of CPPs and mRNA, the mixture was added to approximately 200 nm-sized PLA-NPs (Figure 1(Av)), resulting in nanoparticles with a size of 200–300 nm and a cationic ζ-potential of +25–40 mV. In the case of both CPP/mRNA and PLA-NP/CPP/mRNA complexes, LAH4-L1 provided enhanced reporter protein expression efficiency in DCs compared to that of LAH4, RALA, and a prevalent lipid-based reagent. The His-rich nature of LAH4-L1 may have contributed to the enhanced mRNA introduction efficiency, while the difference between LAH4 and LAH4-L1 might be explained by the steric structure of the peptides [62,63]. Notably, LAH4-L1 induced more efficient protein expression in the presence of PLA-NPs. The advantage of nanoparticle usage could be explained by the larger surface area of the PLA-NP/LAH4-L1/mRNA complex compared with that of the LAH4-L1/mRNA complex interacting with a larger cell surface area and facilitating cellular uptake. Like PPx/GALA complexes, PLA-NP/LAH4-L1/mRNA complexes were mainly endocytosed by clathrin-mediated and phagocytotic pathways rather than macropinocytosis, and the efficiency of mRNA introduction was selective to DCs, with minimal activity observed in HEK293 and HeLa cells. Notably, PLA-NP/LAH4-L1/mRNA complexes activated DCs via mRNA recognition by pattern recognition receptors, Toll-like receptor 3, and retinoic acid-inducible gene-I, and induced the expression of Th1 cytokines with a minimal increase in Th2 cytokine levels. Such Th1-skewed responses are beneficial for preventive vaccines against infectious diseases, as Th2-skewed responses may cause vaccine-enhanced disease.

### 3.4. Lung Surfactant Mimic for Pulmonary Delivery

In a report, a CPP-based mRNA delivery system was optimized for pulmonary delivery via inhalation. Administration of mRNA by inhalation constitutes a promising option for effective treatment of various lung diseases, such as cystic fibrosis, asthma, and lung cancer, owing to its non-invasive nature, increased local drug concentration, and reduced side effects derived from the administration system [64,65,66,67,68,69]. Dry powder formulation offers advantages over liquid aerosols in terms of storage, stability, and sterility [70]. In addition, dry powder inhalers are cheaper and easier to operate than nebulizers. However, formulating mRNA into dry powder aerosols with preserved integrity and biological activity during the drying process is challenging, as long single-stranded mRNAs are fragile and labile to thermal and shear stresses [71,72]. For effective lung deposition, highly dispersible and good aerodynamic properties are needed [73,74,75]. Lam et al. formulated peptide/mRNA complexes as dry powders based on different engineering techniques, such as spray drying (SD) and spray freeze drying (SFD) [48]. In SD, the solution is sprayed to prepare fine droplets, which are then immediately dried using hot gas. In SFD, the sprayed droplets are immediately frozen and then sublimated by freeze-drying.

After pulmonary delivery, naked RNA is effectively internalized by lung cells, presumably by the pulmonary surfactant protein acting as a natural transfection reagent [76,77]. This observation prompted the authors to use KL4, a pulmonary surfactant protein B (SP-B) mimic, for mRNA introduction into the lung. KL4 is an amphipathic CPP composed of Lys and Leu residues (Table 1), demonstrating its potential as a non-viral vector for pulmonary RNA delivery. However, the poor solubility of KL4 hinders its clinical application, and thus 600 Da hydrophilic PEG was covalently attached to KL4 (PEG12KL4) (Figure 1(Avi)). PEG12KL4/mRNA complexes exhibited a size of 300–500 nm with an approximate ζ-potential of +30 mV. After SD, dry powder with a size below 5 μm was prepared, which was able to reach deep into the human lung [75]. In contrast, SFD provided a size larger than 10 μm. Importantly, the integrity and translational capability of the mRNA was preserved after both SD and SFD. The dry powder prepared by SD and SFD induced enhanced reporter protein expression efficiency compared to pulmonary administration of naked mRNA and a lipid-based reagent in liquid form, and was characterized by minor inflammatory responses and low toxicity.

### 3.5. Intracellular mRNA Stabilization

**Figure 2:**
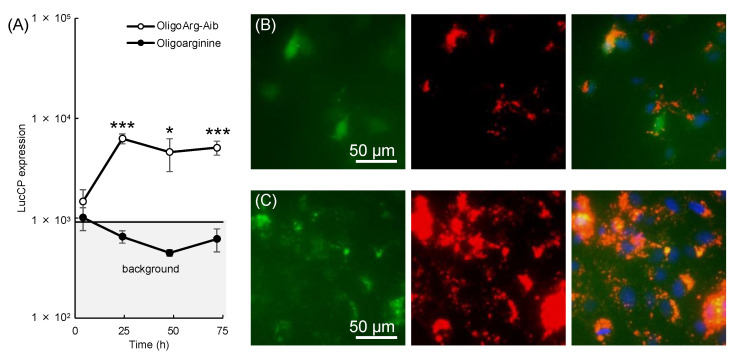
Oligoarginine-α-aminoisobutyric acid (OligoArg-Aib) for cell-penetrating peptides (CPPs) for prolonged protein expression from mRNA. (**A**) Expression of a destabilized reporter protein after its introduction using oligoarginine/mRNA and OligoArg-Aib/mRNA complexes. Statistical analyses were performed using Student’s *t*-test. *, *p* < 0.05, ***, *p* < 0.001. (**B**,**C**) Fluorescence microscopic images of cells 24 h after treatment with oligoarginine/mRNA (**B**) and OligoArg-Aib/mRNA (**C**) complexes. Green: fluorescein-labeled peptides, Red: Cy5-labeled mRNA. Figures are adapted with permission from [49], published by Royal Society of Chemistry, 2021.

Cationic CPP contributes to intracellular mRNA stabilization, which is one of the most challenging issues in mRNA delivery. As mRNA is rapidly degraded inside cells, repeated administration is needed to supply therapeutic proteins for the treatment of cancer and genetic disorders [78,79]. This issue limits the broad application of mRNA therapeutics to disease treatment beyond vaccination. Indeed, the median intracellular half-life of endogenous mRNA is only 9 h [80], and exogenous mRNA is more rapidly degraded within 1–4 h inside cells [81,82,83]. To tackle this problem, we inserted α-aminoisobutyric acid (Aib), the simplest form of an α,α-disubstituted amino acid, into oligoarginine (Table 1) [49]. These OligoArg-Aib peptides form helical structures, even at short lengths [84,85,86,87,88], and we expected that such a change in structure may contribute to stable binding between mRNA and peptides inside the cells and protect the mRNA from intracellular enzymatic degradation. To evaluate intracellular mRNA stability independently of protein stability, we used a destabilized luciferase reporter (dLuc), which has an intracellular half-life of less than 1 h at the protein level. Although dLuc expression from oligoarginine/mRNA complexes became undetectable within 1 day after mRNA introduction into cultured cells, OligoArg-Aib was able to extend dLuc expression to 3 days or longer (Figure 2A). This result suggests that OligoArg-Aib protects the mRNA from degradation inside cells and retains its translational activity for 3 days. To obtain a mechanistic understanding, we observed the intracellular behavior of fluorescein-labeled OligoArg-Aib and Cy5-labeled mRNA. Oligoarginine was shown to be diffusely distributed throughout the cells 24 h after introduction of the mRNA (Figure 2B), which might be attributed to weak binding between oligoarginine and the mRNA. In contrast, OligoArg-Aib exhibited a dotted pattern inside the cells (Figure 2C), which colocalized with the mRNA. This might have reflected the stable binding of OligoArg-Aib to the delivered mRNA. In addition to the use of the OligoArg-Aib/mRNA complex alone, OligoArg-Aib could be used as a building block for the preparation of functionalized mRNA nanoparticles in combination with lipids or polymers. Notably, intracellular stabilization of mRNA using lipid-based systems is challenging as lipids fuse with cellular and endosomal membranes during nucleic acid introduction [89] and thus rarely establish stable interactions with the mRNA in the cytoplasm.

## 4. Future Perspectives

While lipid-based systems are the most advanced mRNA delivery systems, the studies introduced in this review highlight the distinct features of CPPs (Figure 1B). RALA/mRNA complexes yielded an enhanced vaccination effect in vivo compared to standard liposomal mRNA formulations, which may be attributed to the low inflammatory nature of RALA peptides [42]. Nanoparticles coated with GALA or LAH4-L1 were internalized by cells through pathways independent of macropinocytosis [46,47]; whereas macropinocytosis, a dominant pathway in the uptake of lipid-based nanoparticles, was suppressed in mature DCs. As a result, these CPP-based nanoparticles elicited enhanced epitope presentation by DCs compared to mRNA introduction using lipid-based systems, demonstrating their potential for vaccination. Other features of CPPs include their potential to increase the duration of protein expression (Figure 2) [49], which is a challenging task using lipid-based systems.

Meanwhile, the therapeutic application of CPP/mRNA complexes is yet to be demonstrated. For such applications, the physicochemical properties should be tuned to allow safe and efficient in vivo administration. Most of the nanoparticles presented in this review have a size of several hundred nanometers and highly cationic surface charges. Stealth coating of nanoparticles using PEG or other polymers is a potential strategy to reduce the size and surface charge of the nanoparticles. Combinations with other established mRNA delivery systems, such as lipid nanoparticles, present another promising strategy to improve the bioavailability of mRNAs. Imaging of CPP nanoparticles to observe their in vivo functionalities is also an essential step in designing delivery systems. In this regard, CPPs have been utilized to deliver reagents for magnetic resonance imaging and ultrasound imaging, along with therapeutic reagents [90,91]. Employment of such theranostic approaches in mRNA delivery will allow for precise therapy in targeted tissues in the future. Based on the considerable achievements made in mRNA delivery using CPPs, as well as previous successes of CPPs in the delivery of pDNA and siRNA, we believe that CPPs will provide a substantial contribution to the development of mRNA vaccines and therapeutics.

## Figures and Tables

**Table 1:** Summary of representative cell-penetrating peptides for mRNA delivery.

Name	Sequence	Main Functions	Formulation	Ref.
RALA	WEARLARALARALARHLARALARALRACEA	Disruption of endosomal membrane	CPP/mRNA (non-covalent)	[42]
RGSG	WEGRSGRGSGRGSGRHSGRGSGRGSRGCEA	Control		
RRRR	WEGRRRRRRRCEA	Control		
PF14	Stearyl-AGYLLGKLLOOLAAAALOOLL ^(a)^	Disruption of endosomal membrane	CPP/mRNA (non-covalent)	[43]
PFVYLI	PFVYLI	Enhanced cellular uptake	CPP-mRNA (covalent)	[44]
WSYGLRPG	WSYGLRPG	Control		
KALA	WEAKLAKALAKALAKHLAKALAKALKA	Disruption of endosomal membrane	CPP-LNP/mRNA lipoplex	[45]
R8	RRRRRRRR	Control		
GALA	WEAALAEALAEALAEHLAEALAEALEALAA	Modulation of endocytotic pathways in DCs	Polyplex coated with CPP-PEG	[46]
Melittin	GIGAVLKVLTTGLPALISWIKRKRQQ	Control		
LEDE	IGKEFKRIVERIKRFLRELVRPLR	Control		
LAH4-L1	KKALLAHALHLLALLALHLAHALKKA	Modulation of endocytotic pathways in DCs	Nanoparticle coated with CPP/mRNA	[47]
LAH4	KKALLALALHHLAHLALHLALALKKA	Control		
RALA	WEARLARALARALARHLARALARALRACEA	Control		
KL4	KLLLLKLLLLKLLLLKLLLLK	Lung surfactant mimetic	PEG-CPP/mRNA	[48]
OligoArg-Aib	RRXRRXRRXRRXRRX ^(b)^	Intracellular mRNA protection	CPP/mRNA (non-covalent)	[49]
OligoArg	RRRRRRRRR	Control		

^(a)^ O: ornithine, ^(b)^ X: α-aminoisobutyric acid (Aib).

## Data Availability

Not applicable.
